# Pea3 Transcription Factors and Wnt1-Induced Mouse Mammary Neoplasia

**DOI:** 10.1371/journal.pone.0008854

**Published:** 2010-01-22

**Authors:** Rebecca Baker, Claire V. Kent, Rachel A. Silbermann, John A. Hassell, Lawrence J. T. Young, Louise R. Howe

**Affiliations:** 1 Department of Cell and Developmental Biology, Weill Cornell Medical College, New York, New York, United States of America; 2 Strang Cancer Research Laboratory, Rockefeller University, New York, New York, United States of America; 3 Department of Biochemistry and Biomedical Sciences, McMaster University, Hamilton, Ontario, Canada; 4 Center for Comparative Medicine, University of California Davis, Davis, California, United States of America; Roswell Park Cancer Institute, United States of America

## Abstract

The role of the PEA3 subfamily of Ets transcription factors in breast neoplasia is controversial. Although overexpression of PEA3 (E1AF/ETV4), and of the related factors ERM (ETV5) and ER81 (ETV1), have been observed in human and mouse breast tumors, PEA3 factors have also been ascribed a tumor suppressor function. Here, we utilized the MMTV/*Wnt1* mouse strain to further interrogate the role of PEA3 transcription factors in mammary tumorigenesis based on our previous observation that *Pea3* is highly expressed in MMTV/*Wnt1* mammary tumors. *Pea3* expression in mouse mammary tissues was visualized using a *Pea3^NLSlacZ^* reporter strain. In normal mammary glands, *Pea3* expression is predominantly confined to myoepithelial cells. *Wnt1* transgene expression induced marked amplification of this cell compartment in nontumorous mammary glands, accompanied by an apparent increase in *Pea3* expression. The pattern of *Pea3* expression in MMTV/*Wnt1* mammary glands recapitulated the cellular profile of activated β-catenin/TCF signaling, which was visualized using both β-catenin immunohistochemistry and the β-catenin/TCF-responsive reporter *Axin2^NLSlacZ^*. To test the requirement for PEA3 factors in *Wnt1*-induced tumorigenesis, we employed a mammary-targeted dominant negative PEA3 transgene, ΔNPEA3En. Expression of ΔNPEA3En delayed early-onset tumor formation in MMTV/*Wnt1* virgin females (*P* = 0.03), suggesting a requirement for PEA3 factor function for *Wnt1*-driven tumor formation. Consistent with this observation, expression of the ΔNPEA3En transgene was profoundly reduced in mammary tumors compared to nontumorous mammary glands from bigenic MMTV/*Wnt1*, MMTV/ΔNPEA3En mice (P = 0.01). Our data provide the first description of *Wnt1*-mediated expansion of the *Pea3*-expressing myoepithelial compartment in nontumorous mammary glands. Consistent with this observation, mammary myoepithelium was selectively responsive to *Wnt1*. Together these data suggest the MMTV/*Wnt1* strain as a potential model of basal breast cancer. Furthermore, this study provides evidence for a protumorigenic role of PEA3 factors in breast neoplasia, and supports targeting the PEA3 transcription factor family in breast cancer.

## Introduction

The mammalian Ets transcription factor superfamily comprises around 26 proteins characterized by highly related DNA-binding domains containing winged-helix-turn-helix motifs [1]–[3]. Ets factors regulate transcription through binding of this ETS DNA binding domain directly to Ets binding sites in the promoters of target genes. The Ets factor PEA3 (also called E1AF and ETV4) was originally identified through its ability to bind a motif in the polyomavirus enhancer and mediate oncogene-dependent activation, and was subsequently assigned to a subfamily of three closely related Ets proteins: PEA3, ERM (ETV5) and ER81 (ETV1) [4]–[7]. PEA3 family members exhibit high sequence homology within their ETS domains, and also have conserved regulatory domains [6]–[14]. Several PEA3-interacting proteins have been described that function as allosteric regulators and transcriptional coactivators [9], [15], [16]. Additionally, both the activity and expression of PEA3 factors can be regulated by receptor tyrosine kinases through MAP kinase signaling [17], [18]. PEA3 factors are expressed in discrete patterns during embryogenesis, and contribute to branching morphogenesis in epithelia as well as to the establishment of motor-neuronal circuitry [19]–[26]. Functional overlap between individual PEA3 factors is suggested not only by their overlapping target specificities, but also by the modest phenotype of *Pea3* knockout mice [27].

Substantial evidence implicates the PEA3 family in breast neoplasia. Expression of all three family members has been detected in human breast cancer cell lines, and strikingly high expression levels have been identified in oncogene-induced breast tumors in mice [28]–[34]. The reported frequency of *PEA3* overexpression in human breast carcinomas ranges from 22–76% [35]–[40]. Some studies have identified correlations with clinicopathological parameters, including overexpression of human epidermal growth factor receptor 2 (HER2/neu) [35]–[37] and poor prognosis [39], though none of these associations are uniformly supported across studies. *PEA3* overexpression has also been observed in cancers of other organs, including the ovary, lung and gastrointestinal tract [41]–[47], and associations between decreased patient survival and *PEA3* overexpression in ovarian carcinoma, gastric cancer and colorectal carcinoma (CRC) have been reported [44], [46], [47].

A key role for the PEA3 family in carcinogenesis is suggested not only by these expression data, but also by the extensive list of protumorigenic genes that can be transactivated by PEA3 proteins [48]. Foremost among these are the matrix metallopeptidases (MMPs), of which multiple family members are coordinately regulated by PEA3 and AP-1 factors [42], [49], [50]. Additional PEA3 family target genes implicated in neoplasia include; urokinase plasminogen activator (uPA), vimentin, cyclooxygenase-2 (COX-2), Twist, MUC4, WT1, osteopontin, mammaglobin, cyclin D3 and HER2 [31], [34], [51]–[60]. Notably, PEA3 factor target genes are frequently coordinately regulated by PEA3 factors in combination with β-catenin and/or AP-1 factors [31], [42], [50], [53], [55], [57], [61]. Consistent with this, correlations have been observed between expression of *PEA3* and known target genes (e.g. *MMP1*, *MMP7*, *COX-2*) in colorectal tumors [41], [42], [44], [45], which characteristically exhibit increased β-catenin levels. Intriguingly, several of the PEA3 targets identified thus far have established roles in epithelial-mesenchymal transition (EMT), suggesting that PEA3 factors may promote tumor progression through regulation of EMT [51], [55], [62], [63].

The expression and gene regulation data summarized above argue for a protumorigenic role of PEA3 factors. Nevertheless, several lines of evidence contradict this notion. In particular, the demonstration of PEA3-mediated suppression of the *HER2* promoter has engendered the hypothesis that PEA3 might have tumor-suppressor function that could be leveraged for anticancer gene therapy [64]–[66]. In support of this goal, *PEA3* overexpression has been shown to inhibit the growth of *HER2*-overexpressing breast and ovarian tumor xenografts *in vivo* [66], [67], and to reduce *in vitro* invasiveness of a cervical cancer cell line [68]. Additionally, one study identified a positive association between increased patient survival and *PEA3* expression in human breast cancers [38]. Intriguingly, the ability of dietary oleic acid to increase *PEA3* expression and thereby repress *HER2* has been proposed as a key mechanism underlying the protective effect of the “Mediterranean diet” with respect to breast cancer [69].

Controversy concerning the role of PEA3 factors in carcinogenesis prompted us to further examine the role of this transcription factor family in mammary neoplasia, using the MMTV/*Wnt1* mouse model in which tumor formation is driven by mammary-targeted expression of a *Wnt1* transgene. The utility of this model was illustrated by our previous observation of extremely high level *Pea3* expression in tumors from MMTV/*Wnt1* mice [31], and was further supported by data from other investigators establishing a correlation between expression of *PEA3* and PEA3 target genes under conditions of Wnt/β-catenin pathway activation [41], [42], [44], [45]. Using a LacZ-based reporter strain, *Pea3^NLSlacZ^*, to detect endogenous *Pea3* expression, we confirm that *Pea3* is selectively expressed in myoepithelial cells in normal mammary gland [70], and demonstrate that *Wnt1* expression induces selective expansion of the myoepithelial cell compartment in non-tumorous mammary glands, accompanied by an apparent increase in *Pea3* expression. Consistent with this observation of Wnt1-induced expansion of the basal cell layer, we show that β-catenin/TCF signaling is strongly and selectively activated in mammary myoepithelium in adult MMTV/*Wnt1* mice. Early onset tumor formation in MMTV/*Wnt1* mice is significantly delayed by expression of a dominant-interfering PEA3 mutant, and expression of this dominant negative PEA3 transgene is markedly reduced in tumors relative to non-tumorous mammary tissues. Together these data suggest that PEA3 factors are important contributors to Wnt1-induced breast neoplasia, and thus strengthen the rationale for PEA3 family transcription factors as anti-breast cancer targets.

## Materials and Methods

### Ethics Statement

All mice were housed in pathogen-free rooms in filter-topped cages at the Laboratory Animal Research Center at The Rockefeller University or at the New York Blood Center. These facilities are accredited by the Association for Assessment and Accreditation of Laboratory Animal Care, and operate in accordance with Federal (PHS Policy on the Human Care and Use of Animals, Guide for the Use and Care of Laboratory Animals, Animal Welfare Act), State and local laws and regulations. All mice were used in accordance with protocols approved by the Institutional Animal Care and Use Committees of either The Rockefeller University or the New York Blood Center. Mice received food and water *ad libitum*.

### Mouse Strains, Breeding, Tissue Harvesting and Processing

The MMTV/*Wnt1* strain expresses a mammary-targeted *Wnt1* transgene under the control of the mouse mammary tumor virus (MMTV) long terminal repeat. MMTV/*Wnt1* females exhibit hyperplastic mammary glands and develop palpable mammary tumors between 4–12 months of age [71]. An MMTV/*Wnt1* breeding colony was maintained by interbreeding MMTV/*Wnt1* males, obtained from The Jackson Laboratory, with wildtype females.

*Pea3^NLSlacZ^* mice have a bacterial β-galactosidase (β-gal; lacZ) expression cassette “knocked-in” to the endogenous *Pea3* locus, and express β-gal from the targeted allele in a pattern that mimics the spatial and temporal expression of the endogenous *Pea3* allele [19], [32], [70]. This strain thus provides a useful reporter of *Pea3* expression patterns *in vivo*. MMTV/*Wnt1* males and *Pea3^+/NLSlacZ^* females were interbred, and female offspring of all four potential genotypes were retained: wildtype (i.e. *Pea3^+/+^*), MMTV/*Wnt1*, *Pea3^+/NLSlacZ^*, and bigenic MMTV/*Wnt1*, *Pea3^+/NLSlacZ^*. Mammary glands and tumors were harvested post-mortem and stained with X-gal to detect β-gal activity, as previously described [72]. Abdominal (#4) mammary glands were wholemounted after staining. Axillary (#3) mammary glands were fixed in formalin, embedded in paraffin, and 8 µm tissue sections were counterstained with eosin.

The *Axin2^NLSlacZ^* strain was employed as a reporter of *in vivo* β-catenin/TCF signaling. *Axin2* is upregulated in response to canonical Wnt/ β-catenin signaling, and functions as a negative feedback regulator [73], [74]. *Axin2^NLSlacZ^* mice have a β-gal expression cassette “knocked-in” to the endogenous *Axin2* locus, and hence provide a useful reporter of *in vivo* β-catenin/TCF pathway activation [74]. *Axin2^+/NLSlacZ^* mice were interbred with MMTV/*Wnt1* animals and tissues were harvested and processed as described for the *Pea3^NLSlacZ^* experiment.

MMTV/ΔNPEA3En mice express a dominant interfering mutant in which the repression domain from the *Drosophila melanogaster* Engrailed (En) protein is appended to the C-terminal DNA-binding domain of PEA3 [32]. Both MMTV/*Wnt1* and MMTV/ΔNPEA3En animals were maintained on an FVB strain background. MMTV/*Wnt1* males and MMTV/ΔNPEA3En females were interbred to generate MMTV/*Wnt1* (n = 19) and bigenic MMTV/*Wnt1*, MMTV/ΔNPEA3En (n = 22) female offspring. Test animals were palpated twice weekly to detect mammary tumors, and the age at first tumor detection was recorded for each animal. Animals were sacrificed when tumors were 1 cm in diameter. Mammary glands and tumors were harvested post-mortem, snap-frozen in liquid nitrogen, and stored at −80°C.

### Mouse Genotyping

Mice were genotyped by polymerase chain reaction (PCR) of tail-tip-derived genomic DNA. The PCR primers used to detect the *Wnt1* transgene were as previously described [31]. *Pea3* wildtype and *Pea3^NLSlacZ^* alleles were detected using the following primer pairs: *Pea3*, 5′-GGA ATC TTG GGC CTT GAG AAC AGC-3′ and 5′-GTG TGA TGT ACA TAT GCC CTA ACC-3′ (686 bp product); *Pea3^NLSlacZ^*, 5′-CAG CCT CTG TTC CAC ATA CAC TCC-3′ and 5′-TAG TAT CGC AGC GAG CGG CTC AGC-3′ (479 bp product). The ΔNPEA3En transgene was detected using one primer each from the *Pea3* gene and the *Engrailed* gene: 5′-TTG AAC CTG AAG AGG TTG CC-3′ and 5′-TGT GGA AAC TCA TGT CAC CG-3′ (727 bp product). The *Axin2* wildtype allele was detected using the following primers: Cs, 5′-AAG CTG CGT CGG ATA CTT GAG A-3′; and Cwt, 5′-AGT CCA TCT TCA TTC CGC CTA GC-3′ (493 bp product). The *Axin2^NLSlacZ^* allele was detected using Cs primer in combination with a LacZ-specific primer 5′-TGG TAA TGC TGC AGT GGC TTG-3′ (∼400 bp product).

### β-Catenin Immunohistochemistry

β-catenin immunohistochemistry (IHC) was performed as previously described [75]. Briefly, after treating tissue sections with hydrogen peroxide to block endogenous peroxidase activity, sections underwent sodium citrate/microwave oven-based antigen retrieval. Slides were incubated overnight with primary antibody (anti-ß-catenin, Clone 14, BD Transduction Labs) and subsequently with secondary antibody for 1 hour (biotinylated horse anti-mouse IgG, Vector Labs). Antigen was visualized using Vectastain ABC reagent followed by 3,3′-diaminobenzidine staining, and a final hematoxylin counterstain. Duplicate slide staining with primary antibody omitted was routinely performed to provide a negative control.

### Reverse Transcription-Coupled PCR (RT-PCR) Analysis

RT-PCR was used to compare gene expression in mammary tissues harvested from five animals each that were MMTV/*Wnt1*, MMTV/ΔNPEA3En and bigenic MMTV/*Wnt1*, MMTV/ΔNPEA3En. Total RNA was prepared from resected, snap-frozen mammary glands and tumors, and cDNA was generated by reverse transcription as previously described [76]. “Mock cDNA” was generated by performing reactions in parallel with those used to generate cDNA, omitting reverse transcriptase enzyme from the reaction. PCR was performed using primers for the *Wnt1* and ΔNPEA3En transgenes, and for RNA Polymerase II (RPII) as a normalization and quality control. Primer sequences were: *Wnt1*, previously described [31]; ΔNPEA3En, 5′-AAG GAG GAG GAA AGC GAC TC-3′ and 5′-AGG AGA TGG CTG CTG AGT TGG-3′; RPII, 5′-GCT TAC CAT GGA ACA GAT TGC TG-3′ and 5′-CAC CTC TTC CTC CTC TTG CAT C-3′. PCR reaction products were fractionated on agarose gels and gel images were captured using a BioRad Molecular Imager ChemiDoc XRS System. No detectable PCR products were generated in reactions using “mock cDNA”. Band intensities were quantitated by analysis on an Apple computer using the public domain NIH Image program (developed at the U.S. National Institutes of Health and available on the Internet at http://rsb.info.nih.gov/nih-image/). Values obtained were normalized to those obtained for RPII. To compare ΔNPEA3En expression in non-tumorous mammary glands and mammary tumors from bigenic MMTV/*Wnt1*, MMTV/ΔNPEA3En animals, the ΔNPEA3En signal was normalized to the *Wnt1* signal.

### Statistical Analysis

Kaplan-Meier curves of tumor-free survival in MMTV/*Wnt1* and bigenic MMTV/*Wnt1*, MMTV/ΔNPEA3En cohorts were compared using a one-tailed log-rank test (Prism 4.0 for Macintosh, GraphPad Software Inc., San Diego, CA). Early onset tumor latency in the two cohorts was compared by censoring survival data at 130 days. Simple independent one-tailed t-tests were performed to effect pairwise comparisons of gene expression between groups (Microsoft Excel 2004 for Mac, Microsoft Corporation, Redmond, WA).

## Results

### *Wnt1* Expression in Mouse Mammary Tissues Drives Expansion of the *Pea3*-Expressing Myoepithelial Population

We have previously identified *Pea3* as being extremely highly expressed in mammary tumors from MMTV/*Wnt1* transgenic mice. Specifically, Northern blotting revealed that *Pea3* was expressed at a similar level to the highly expressed *glyceraldehyde-3-phosphate dehydrogenase* (*GAPDH*) gene utilized as a normalization control [31]. We interpreted this high level *Pea3* expression as potentially indicative of a protumorigenic role for Pea3, particularly given that Pea3 positively regulates transcription of multiple proneoplastic genes [31], [34], [42], [48]–[60]. However, our earlier study did not establish whether *Pea3* is upregulated prior to the formation of frank tumors, since Northern blotting was insufficiently sensitive to detect *Pea3* transcripts in mammary glands prior to tumor development from either wildtype or MMTV/*Wnt1* mice. Therefore, in the present study we utilized a reporter strain, *Pea3^NLSlacZ^*, in which a bacterial β-galactosidase expression cassette is inserted into the endogenous *Pea3* allele [19], [32], [70], to assess *Pea3* expression levels in non-tumorous mammary tissue. The *Pea3^NLSlacZ^* reporter allele has been shown to recapitulate the temporospatial expression patterns previously described for the endogenous *Pea3* allele, and thus appears to provide faithful and sensitive reporting of expression of the endogenous *Pea3* gene [19], [32], [70].

**Figure 1:**
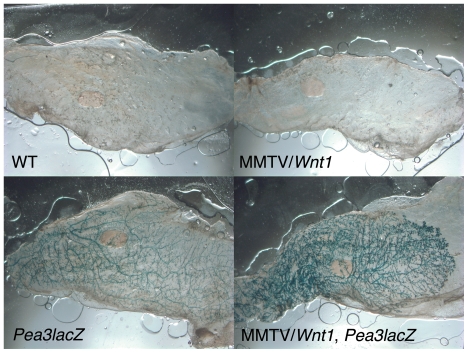
The *Pea3^NLSlacZ^* strain acts as a reporter of *in vivo Pea3* expression in mammary glands. Abdominal (#4) mammary glands were harvested from female mice that were wildtype (WT), MMTV/*Wnt1*, *Pea3^+/NLSlacZ^* (*Pea3lacZ*), and bigenic MMTV/*Wnt1*, *Pea3^+/NLSlacZ^* (MMTV/*Wnt1*, *Pea3lacZ*). Mammary glands were stained with X-gal as previously described [72] and wholemounted. Staining of epithelial structures was observed in mammary glands from *Pea3^+/NLSlacZ^* females and bigenic MMTV/*Wnt1*, *Pea3^+/NLSlacZ^* females, but not in tissues from wildtype or MMTV/*Wnt1* animals.

**Figure 2:**
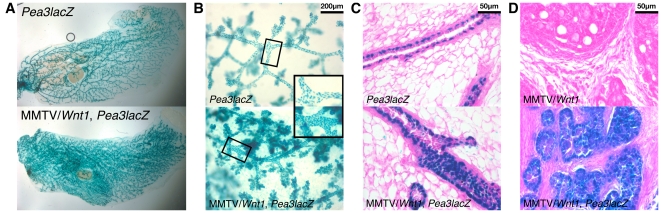
*Wnt1* expression in mammary tissues drives expansion of the *Pea3*-expressing myoepithelial population. Mammary tissues were harvested from age-matched virgin female littermates and stained with X-gal. (**A**) Wholemounted glands (10 weeks old)–entire gland. (**B**) Wholemounted glands (30 weeks old) viewed at 10× magnification (inset, enlargement of boxed area). (**C**) Mammary gland tissue sections (10 weeks old), counterstained with eosin, viewed at 40× magnification. (**D**) Tumor tissue sections, counterstained with eosin, viewed at 40× magnification. β-gal activity, indicative of *Pea3* expression, was detected primarily in myoepithelial cells in normal mammary glands. The frequency of *Pea3*-expressing cells was increased in *Wnt1*-expressing mammary glands, and there was an apparent increase in signal intensity on a per cell basis. *Pea3* was widely expressed throughout the tumor epithelium in MMTV/*Wnt1* mammary tumors.

*Pea3^+/NLSlacZ^* mice were crossed to MMTV/*Wnt1* mice, and mammary glands from female offspring of all four resultant genotypes were stained with X-gal to detect β-gal activity. Distinct staining of epithelial structures was apparent in wholemounted mammary glands from *Pea3^+/NLSlacZ^* females and bigenic MMTV/*Wnt1*, *Pea3^+/NLSlacZ^* females, but was not observed in tissues from animals lacking the *Pea3^NLSlacZ^* allele (Figure 1). Gross examination of X-gal-stained wholemounted glands from age-matched *Pea3^+/NLSlacZ^* and bigenic MMTV/*Wnt1*, *Pea3^+/NLSlacZ^* virgin females suggested that *Wnt1* expression was associated with increased signal intensity (Figure 2A). Microscopic examination of both wholemounts and tissue sections revealed that β-gal activity, and hence *Pea3* expression, was restricted predominantly to the myoepithelial cells comprising the outer epithelial layer of the mammary ducts (Figures 2B and C), as previously reported [70]. Sporadic β-gal-positive nuclei were also evident in nerve bundles within the mammary glands (data not shown). Unexpectedly, we observed that the number of *Pea3*-positive myoepithelial cells was increased in *Wnt1*-expressing mammary ducts (Figures 2B and C), suggesting that the myoepithelial compartment is selectively amplified in response to *Wnt1* expression. Additionally, the signal intensity in individual myoepithelial cells appeared to be increased in bigenic MMTV/*Wnt1*, *Pea3^+/NLSlacZ^* mammary glands relative to that in *Pea3^+/NLSlacZ^* samples (Figures 2B and C).

### *Pea3* Is Highly Expressed in MMTV/*Wnt1* Tumor Epithelium

The extremely high level of *Pea3* expression in MMTV/*Wnt1* mammary tumors rendered it technically challenging to reliably achieve X-gal staining of tumor samples, due to rapid precipitation of reaction product on the tissue surface. This problem was partially overcome by cutting tumor tissue samples into multiple slices prior to staining, and carefully sectioning the outermost face in the plane of the gross slice. Using this approach, we found that *Pea3* expression was predominantly restricted to, and highly expressed in, the tumor epithelium (Figure 2D), which contains cells of both myoepithelial and luminal lineages [77]–[79]. Widespread expression throughout the tumor epithelium was confirmed by *in situ* hybridization analysis (R Baker and LR Howe; unpublished data).

### β-Catenin/TCF Signaling Is Preferentially Activated in the Myoepithelial Compartment in MMTV/*Wnt1* Mammary Glands

**Figure 3:**
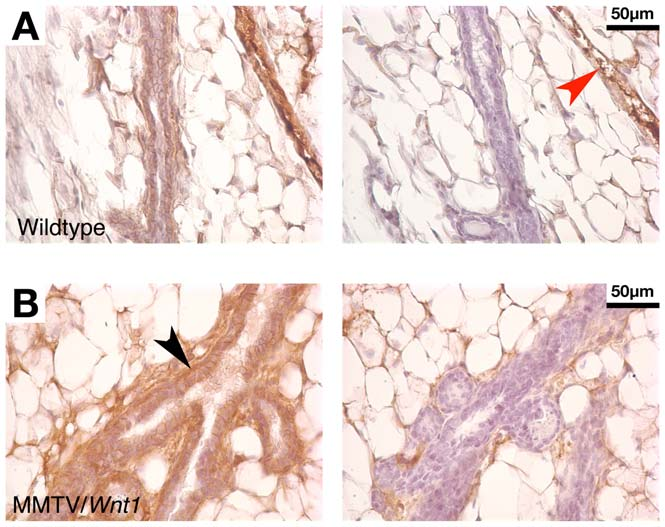
*Wnt1* expression in mouse mammary glands induces profound β-catenin accumulation in the myoepithelial compartment. Mammary gland tissue sections from virgin female mice (10 weeks old) were subjected to β-catenin IHC as previously described [75] and counterstained with hematoxylin. As a control, serial sections were stained in parallel omitting primary antibody (right-hand panels). (**A**) Wildtype. (**B**) MMTV/*Wnt1*. Weak staining of membrane-localized β-catenin was observed in wildtype epithelium. Vascular structures were clearly evident in control slides stained with secondary antibody alone (red arrowhead). Profound increases in β-catenin protein levels were observed in MMTV/*Wnt1* epithelium, including both increased membrane signal as well as β-catenin accumulation in the cytoplasm and nucleus. Cells in the myoepithelial compartment exhibited the strongest β-catenin signal (black arrowhead).

To further investigate the unexpected finding that the myoepithelial compartment was selectively amplified in MMTV/*Wnt1* mammary glands, we sought to define which cell types exhibit activation of the canonical Wnt/β-catenin signaling pathway in response to *Wnt1* transgene expression. Our first approach was to perform β-catenin immunohistochemistry (IHC) to visualize nucleocytoplasmic accumulation of β-catenin protein, the hallmark of canonical Wnt signaling. As expected, β-catenin protein was localized to the plasma membrane of epithelial cells in wildtype mouse mammary glands, consistent with its role at the adherens junction (Figure 3A). There was a marked increase in β-catenin protein levels in premalignant mammary tissues from MMTV/*Wnt1* mice (Figure 3B). Not only was increased membrane staining detected, but there was also substantial accumulation of nucleocytoplasmic β-catenin protein. Notably, the most robust staining was observed in the myoepithelial cell layer (Figure 3B; black arrowhead).

**Figure 4:**
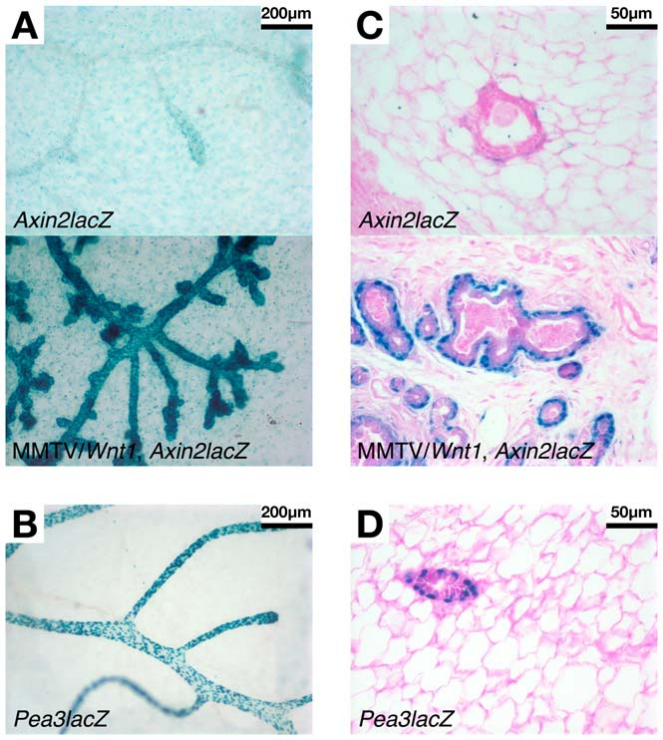
β-catenin/TCF signaling activity revealed using *Axin2^NLSlacZ^* mice mirrors nucleocytoplasmic β-catenin accumulation. Abdominal (#4) mammary glands were harvested from female mice that were *Axin2^+/NLSlacZ^* (*Axin2lacZ*) or bigenic MMTV/*Wnt1*, *Axin2^+/NLSlacZ^* (MMTV/*Wnt1*, *Axin2lacZ*), and were stained with X-gal as previously described [72]. *Pea3^+/NLSlacZ^* (*Pea3lacZ*) samples were also included for comparison. (**A**) Wholemounted glands (11 weeks old), viewed at 10× magnification. (**B**) Wholemounted gland from *Pea3lacZ* virgin female stained in parallel with the specimens in Panel A, viewed at 10× magnification. (**C**) Mammary gland tissue sections (11 weeks old), counterstained with eosin, viewed at 40× magnification. (**D**) Mammary gland tissue section from *Pea3lacZ* virgin female (10 weeks old), counterstained with eosin, viewed at 40× magnification. β-gal activity was detected in adipocytes throughout the mammary fat pad from both *Axin2^+/NLSlacZ^* and bigenic MMTV/*Wnt1*, *Axin2^+/NLSlacZ^* mice. Additionally, in *Axin2^+/NLSlacZ^* mammary gland, cells in the connective tissue peripheral to the mammary epithelium stained positive, but no staining was detected in the myoepithelial or luminal cells. *Wnt1* expression induced robust β-gal activity, indicative of β-catenin/TCF signaling, in the myoepithelial compartment, with weaker but detectable signal in the luminal epithelium.

As a corollary approach, the pattern of expression of a β-catenin/TCF-responsive gene, *Axin2*, was compared in wildtype and *Wnt1*-expressing mammary glands using the *Axin2^NLSlacZ^* reporter strain. Transcriptional activation of *Axin2* appears to be a widespread, if not ubiquitous, response to canonical Wnt/β-catenin signaling [73], [74], and hence *Axin2* transcripts are increasingly used as a convenient readout of β-catenin/TCF pathway activation. The *Axin2^NLSlacZ^* mouse strain has a β-gal expression cassette “knocked-in” to the endogenous *Axin2* locus, and thus provides a useful tool for visualizing β-catenin/TCF signaling *in vivo* [74]. MMTV/*Wnt1* mice were crossed with *Axin2^+/NLSlacZ^* animals, and tissues from virgin female offspring of the four resulting genotypes were stained with X-gal. As in the *Pea3lacZ* experiment described above, β-gal activity was undetectable in mammary glands from wildtype or MMTV/*Wnt1* animals (data not shown). In contrast, β-gal signal was detected in adipocytes throughout the fat pad of mammary glands from both *Axin2^+/NLSlacZ^* mice and bigenic MMTV/*Wnt1*, *Axin2^+/NLSlacZ^* littermates (Figure 4A). Similar observations were also made using two additional TCF reporter strains (BAT-gal and TOPGAL; S Takayama, AP Salmon and LR Howe; unpublished data) suggesting that there may be constitutive Wnt/β-catenin signaling in mammary adipocytes. However, consistent with the lack of nucleocytoplasmic β-catenin in wildtype mammary epithelium (Figure 3A), no β-gal activity was detected in the mammary epithelium of *Axin2^+/NLSlacZ^* mice in the absence of the *Wnt1* transgene (Figures 4A and C; *Axin2lacZ*). Positively staining fibroblast nuclei were observed in the connective tissue peripheral to the mammary epithelium, but no staining was detected in the luminal or myoepithelial cells (Figure 4C; *Axin2lacZ*), indicating that there is little or no active Wnt/β-catenin signaling in the mammary epithelium at this stage. This contrasts with the robust myoepithelial staining observed in *Pea3^+/NLSlacZ^* mammary gland in the absence of *Wnt1* expression (Figures 4B and D), and thus suggests that *Pea3* expression in normal mammary gland may be regulated by non-Wnt signals.

Expression of the *Wnt1* transgene induced profound activation of *Axin2lacZ* expression, a surrogate for β-catenin/TCF signaling. Mammary epithelium from bigenic MMTV/*Wnt1*, *Axin2^+/NLSlacZ^* mice exhibited intense X-gal staining which was most prominent in the myoepithelial layer (Figures 4A and C; MMTV/*Wnt1*, *Axin2lacZ*) [80]. Additionally, weaker but detectable staining was observed in the inner, luminal epithelium in *Wnt1*-expressing glands (Figure 4C). The finding of prominent lacZ expression in the myoepithelial compartment in MMTV/*Wnt1*, *Axin2^+/NLSlacZ^* mammary glands is in striking concordance with the strong nucleocytoplasmic β-catenin staining observed in this cell layer (Figure 3B), and also mirrors the X-gal staining pattern in bigenic MMMTV/*Wnt1*, *Pea3^+/NLSlacZ^* mammary glands (Figures 2B and C).

### Dominant Negative PEA3 Suppresses Tumor Formation in MMTV/*Wnt1* Mice

Based on our observations of *Pea3* upregulation in *Wnt1*-expressing mammary glands and tumors [31] (Figure 2), we sought to assess the requirement for PEA3 factors for Wnt1-induced tumorigenesis. This question was addressed using a previously generated dominant negative transgenic mouse strain MMTV/ΔNPEA3En [32]. The ΔNPEA3En allele encodes a fusion protein consisting of the repressor domain from the *Drosophila* Engrailed protein appended to the PEA3 C-terminus, which encompasses the DNA-binding domain. The resultant mutant protein lacks the PEA3 activation domain, and hence functions as a dominant negative by binding to cognate PEA3 sites and repressing transcription through the En repressor domain. This construct has been shown to antagonize transcriptional activation by all three PEA3 family members; PEA3, ERM, and ER81 [32], which have overlapping target specificity. This is important because all three PEA3 family proteins are expressed in MMTV/*Wnt1* mammary tumors [31]. Use of the ΔNPEA3En dominant negative allele circumvents potential functional redundancy between the PEA3 family proteins, and thus offers a more robust approach than a gene knockout strategy targeting individual PEA3 factors. Conversely, one caveat with respect to this strategy is potential lack of specificity of ΔNPEA3En. However, although the spectrum of activity of this dominant-interfering PEA3 allele is not fully defined, it fails to alter expression from the MMTV promoter [32], which contains an Ets-binding site regulated by the Ets protein GABP-α, indicating that only a subset of Ets factors are susceptible to ΔNPEA3En-mediated repression.

**Figure 5:**
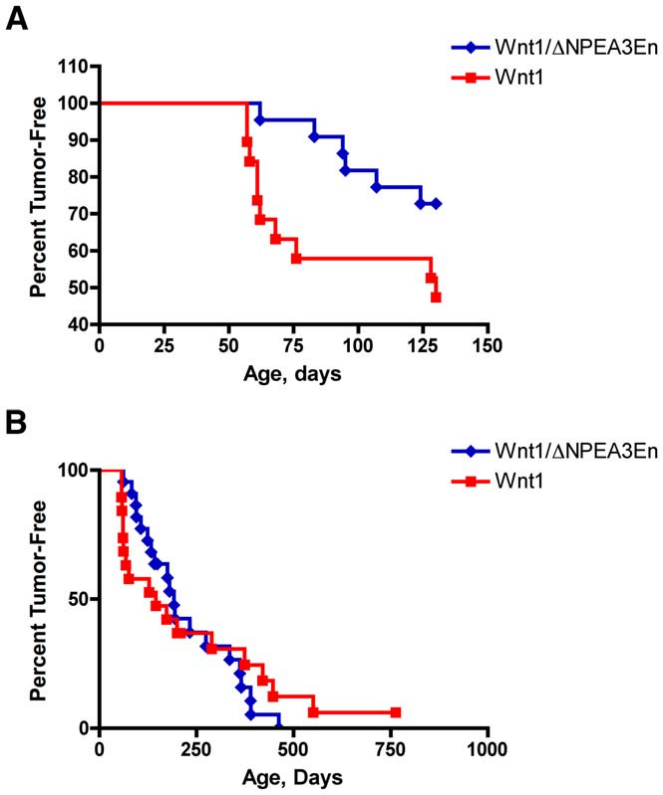
The dominant-interfering ΔNPEA3En transgene suppresses early onset tumor formation in MMTV/*Wnt1* mice. Tumor latency was compared in two cohorts of virgin female mice, MMTV/*Wnt1* (red squares; n = 19) and bigenic MMTV/*Wnt1*, MMTV/ΔNPEA3En (blue diamonds; n = 22), generated by interbreeding MMTV/*Wnt1* males with MMTV/ΔNPEA3En females. (**A**) Formation of early onset tumors was substantially retarded by expression of the dominant-interfering ΔNPEA3En transgene (*P* = 0.03, log-rank test). Survival data were censored at 130 days to compare early onset tumor formation (**B**) The overall rate of tumor formation was similar in both cohorts, suggesting that tumors were ultimately able to escape from ΔNPEA3En-mediated tumor suppression (*P* = 0.39, log-rank test).

To examine the effect of dominant negative PEA3 on Wnt1-driven tumorigenesis, we compared tumor latency in MMTV/*Wnt1* and bigenic MMTV/*Wnt1*, MMTV/ΔNPEA3En virgin females, generated by interbreeding MMTV/*Wnt1* mice with MMTV/ΔNPEA3En animals. Expression of the dominant-interfering PEA3 mutant significantly delayed the formation of early onset tumors in MMTV/*Wnt1* mice (Figure 5A; *P* = 0.03). To exclude the possibility that this effect was due to ΔNPEA3En-mediated suppression of expression of the tumor-driving *Wnt1* oncogene, *Wnt1* expression was compared in MMTV/*Wnt1* and bigenic MMTV/*Wnt1*, MMTV/ΔNPEA3En tissues. Co-expression of MMTV/ΔNPEA3En did not significantly decrease *Wnt1* expression, assayed by RT-PCR, in either mammary glands or tumors (Table 1). Thus this experiment demonstrates that dominant negative PEA3 attenuates Wnt1-induced mammary neoplasia.

**Table 1:** *Wnt1* transgene expression is not significantly altered by coexpression of ΔNPEA3En.

Tissue Type	Genotype	*Wnt1* Transgene Expression^a^ (Mean ± SD)	*P* b
Mammary gland	MMTV/*Wnt1*	1.27±0.64	-
	MMTV/*Wnt1*, MMTV/ΔNPEA3En	0.95±0.49	0.11
Mammary tumor	MMTV/*Wnt1*	1.81±1.09	-
	MMTV/*Wnt1*, MMTV/ΔNPEA3En	1.52±0.71	0.30

*Wnt1* transcripts were assayed by RT-PCR in mammary glands and tumors from MMTV/*Wnt1* and bigenic MMTV/*Wnt1*, MMTV/ΔNPEA3En animals (n = 5 per group). *Wnt1* transcript levels were normalized to RNA Polymerase II (RPII) as an internal control.

a Normalized to RPII.

b *P* value obtained by comparison of values in MMTV/*Wnt1* and bigenic MMTV/*Wnt1*, MMTV/ΔNPEA3En tissues, using a one-tailed t-test.

**Figure 6:**
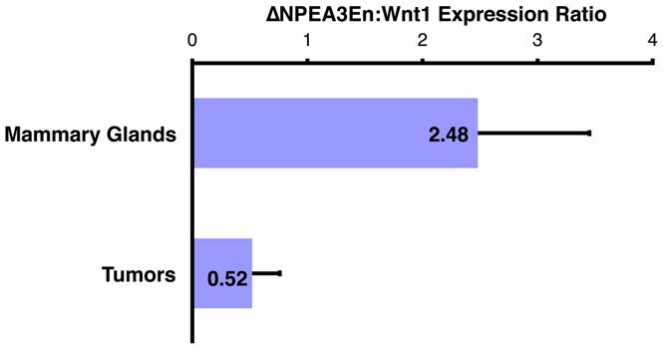
ΔNPEA3En expression is markedly decreased in tumors relative to mammary glands from MMTV/*Wnt1*, MMTV/ΔNPEA3En mice. Expression levels of the *Wnt1* and ΔNPEA3En transgenes in mammary glands and tumors from bigenic MMTV/*Wnt1*, MMTV/ΔNPEA3En mice were assayed by RT-PCR. The expression level of ΔNPEA3En in each sample was normalized to that of *Wnt1* in the same sample to control for potential variations in epithelial content between tumors and nontumorous mammary glands. The ratio of ΔNPEA3En expression to *Wnt1* expression was strikingly reduced in mammary tumors relative to non-tumorous mammary glands (*P* = 0.01, t-Test).

The median time to tumor formation was 145 days in MMTV/*Wnt1* mice compared to 192 days in those animals also bearing the ΔNPEA3En transgene. However, the Kaplan-Meier survival curves intersected at 274 days (Figure 5B), suggesting that tumors were ultimately able to “escape” suppression mediated by dominant negative PEA3. One potential explanation for this escape phenomenon is loss of expression of the ΔNPEA3En transgene. To investigate this possibility, expression levels of the ΔNPEA3En transgene in mammary glands and tumors were measured by RT-PCR. The ΔNPEA3En transgene is expressed from the MMTV promoter, which is primarily active in epithelial cells in mouse mammary tissues. MMTV-regulated transcripts can appear artificially elevated in tumors relative to premalignant mammary gland when expression is normalized to a “housekeeping” gene expressed in both epithelial and stromal compartments because the ratio of epithelium to stroma is increased in tumors compared with non-tumorous mammary glands. To control for this potential artefact in measuring ΔNPEA3En transgene expression, ΔNPEA3En levels were normalized to those of the *Wnt1* transgene, which is also expressed from the MMTV promoter. Strikingly, the ΔNPEA3En:*Wnt1* expression ratio was substantially reduced in mammary tumors relative to non-tumorous mammary glands from bigenic MMTV/*Wnt1*, MMTV/ΔNPEA3En mice (Figure 6; *P* = 0.01). These data strongly suggest that suppression of ΔNPEA3En expression is a prerequisite for tumor formation in bigenic MMTV/*Wnt1*, MMTV/ΔNPEA3En animals, and thus provide additional evidence for an important role of PEA3 factors in Wnt1-mediated mammary tumorigenesis. Interestingly, a similar phenomenon was reported by Shepherd *et al.* (2001) who crossed the ΔNPEA3En transgenic with MMTV/*neu* mice and identified reduced expression of the ΔNPEA3En transgene in tumors relative to mammary glands [32]. The molecular mechanism underlying loss of ΔNPEA3En expression was not identified, but could potentially involve loss of the transgene from the genomic DNA or epigenetic silencing of the transgene locus.

## Discussion

We and others have shown that *Pea3* is highly expressed in mouse breast tumors, and *PEA3* overexpression also occurs in human breast carcinomas [30]–[33], [35]–[40], [81]. Furthermore, Pea3 activates transcription of multiple neoplasia-associated genes, including *MMP* genes, *COX-2*, *Twist*, *osteopontin*, *uPA*, *vimentin*, *MUC4*, *WT1*, *mammaglobin*, *cyclin D3* and *HER2* [31], [34], [42], [48]–[60]. For example, we have shown PEA3 to be a potent activator of *COX-2* transcription, and in turn have shown Cox-2 to be an important contributor to HER2/neu-induced mammary neoplasia and angiogenesis in mouse models [31], [76], [82]. Nevertheless, other investigators have argued in favor of PEA3 functioning as a tumor suppressor, based in part on the ability of high level *PEA3* expression to suppress *HER2* promoter activity *in vitro* and *HER2*-expressing tumor growth *in vivo* [64], [66], [67], [69]. Consistent with a tumor-promoting function for PEA3, one study identified a positive association between increased patient survival and *PEA3* expression in human breast cancers [38].

In order to further assess the role of PEA3 factors in mammary tumorigenesis, we chose a model in which breast neoplasia was not driven by *HER2/neu* overexpression. We employed the MMTV/*Wnt1* strain because we had previously established that MMTV/*Wnt1* tumors exhibit robust *Pea3* expression [31]. The evidence presented here suggests a protumorigenic role for PEA3 factors in Wnt1-induced mammary neoplasia. Specifically, the dominant-interfering mutant ΔNPEA3En suppressed early onset tumor formation in MMTV/*Wnt1* mice (Figure 5A), and ΔNPEA3En expression was markedly suppressed in those tumors that did develop in bigenic MMTV/*Wnt1*, MMTV/ΔNPEA3En animals (Figure 6). These data, which support a positive role of PEA3 factors in breast neoplasia, are consistent with those obtained by other groups using a variety of approaches. Several studies have shown that enhanced invasiveness, cell cycle progression and tumorigenicity can be conferred by overexpressing *PEA3* in breast or lung cancer cell lines [43], [56], [83]. Conversely, suppressing the expression or activity of PEA3 factors has been shown to reduce proliferation and invasiveness of cancer cell lines in culture, and to decrease *in vivo* tumorigenesis [32], [48], [84]. Furthermore, RNAi-mediated knock-down of *Pea3* in mouse mammary tumor cells leads to decreased expression of multiple PEA3 gene targets with established proneoplastic roles, including *Cox-2*, *vimentin*, *Cyclin D3*, *HER2/neu* and several *MMP* genes [48]. Of note, positive correlations between poor prognosis and *PEA3* overexpression have been identified in cancers of several tissue sites including carcinomas of the breast, ovary, stomach and CRC [39], [44], [46], [47]. In aggregate, these data support the potential therapeutic utility of targeting PEA3 factors.

Our data provide a novel proof-of-principle that neoplasia resulting from activation of Wnt/β-catenin signaling can be attenuated by suppressing Ets factor activity. This finding may also be relevant in the context of colorectal cancer, which is frequently driven by mutational activation of the Wnt/β-catenin pathway. Importantly in this respect, PEA3 factor overexpression is prevalent both in human colorectal cancers and in intestinal tumors from mouse CRC models [41], [42], [44], [45]. Thus, we predict that targeting PEA3 factors may also be of therapeutic benefit for colorectal cancer patients.

Intriguingly, the interrelationship between Wnt/β-catenin signaling and PEA3 may not be restricted to neoplasia, but may also be important during normal mammary morphogenesis. Potential functional interaction between PEA3 and Wnt signaling during postnatal mammary development is suggested by the overlapping phenotypes elicited by *Pea3* nullizygozity and by genetic ablation of *LRP5* (low density lipoprotein receptor related protein 5), a key component of the Wnt receptor complex. Thus, both *Pea3*-null and *LRP5*-deficient mice exhibit a delay in terminal end-bud formation and regression during mammopoiesis [70], [85].

In addition to determining the role of PEA3 factors in Wnt1-induced tumorigenesis, we also sought to characterize the effect of Wnt1 on *Pea3* expression in non-tumorous mammary glands. Analysis of the LacZ-based reporter strain *Pea3^NLSlacZ^* revealed an apparent increase in intensity of *Pea3* expression in mammary myoepithelial cells in response to Wnt1 (Figure 2). Additionally, this experiment revealed an unanticipated consequence of *Wnt1* expression in mouse mammary gland. Specifically, Wnt1 stimulates expansion of the myoepithelial compartment prior to tumor formation (Figure 2). Several groups have previously characterized MMTV/*Wnt1* tumors as containing both myoepithelial and luminal epithelial cells, based on the presence of discrete cell populations exhibiting myoepithelial and luminal markers, and have interpreted this as evidence that Wnt1 acts on multipotent progenitor cells in mouse mammary glands [77]–[79]. Furthermore, Wnt1-induced mammary hyperplasia has been theorized as resulting from accumulation of relatively undifferentiated progenitor and transit-amplifying cells [86]. The observation that Wnt1 induces specific amplification of the myoepithelial, or basal, compartment in *Wnt1*-expressing mammary gland prior to tumor formation suggests that these putative Wnt1-responsive progenitor cells may reside in the basal cell layer.

To further investigate the observation that Wnt1 induces amplification of the mammary myoepithelial compartment, we characterized the cellular response profile to *Wnt1* expression using both β-catenin IHC and a β-catenin/TCF-responsive reporter strain, *Axin2^NLSlacZ^*. β-catenin immunostaining revealed a profound increase in β-catenin protein in MMTV/*Wnt1* mammary glands relative to the modest membrane signal in wildtype tissues (Figure 3). Of particular note, we observed intense nucleocytoplasmic signal in the myoepithelial cell layer. Consistent with this immunohistochemical staining pattern, there was profound induction of the β-catenin/TCF-responsive reporter *Axin2^NLSlacZ^* in *Wnt1*-expressing mammary epithelium (Figure 4). The strongest signal was evident in the basal layer, as recently reported [80]. Additionally, we detected lower signal intensity in the inner, luminal layer. However, no epithelial *Axin2^NLSlacZ^* activity was detected in adult mammary glands in the absence of *Wnt1* transgene expression. This contrasts with our findings of *Pea3* expression in mammary myoepithelium in the absence of *Wnt1*, and suggests that *Pea3* expression in normal adult mammary gland is regulated by non-Wnt signals. Interestingly, β-catenin/TCF signaling activity was observed in mammary adipocytes irrespective of the presence of the *Wnt1* transgene, as revealed by expression of *Axin2^NLSlacZ^* (Figure 4A and C) and also of other β-catenin/TCF-responsive reporter alleles (BAT-gal and TOPGAL; S Takayama, AP Salmon and LR Howe; unpublished data). The significance of this observation is unclear since Wnt/β-catenin signaling is well characterized as a negative regulator of adipogenesis [87]. Nevertheless, it is clear that *Wnt1* transgene expression induces a profound β-catenin/TCF signaling response in mammary myoepithelial cells, which mirrors the *Pea3^NLSlacZ^* staining pattern in MMTV/*Wnt1* mammary glands (compare Figures 3B and 4C with 2C). Together these datasets suggest that the myoepithelial compartment is preferentially responsive to Wnt1. The selective Wnt1-responsiveness of the myoepithelium, and the fact that Wnt1 induces expansion of this cell layer, most likely reflects restricted expression of functional Wnt receptor complexes since *LRP6* expression is limited to basal cells in adult mouse mammary gland [88]. Consistent with these observations, a basal gene signature has been identified in cells extracted from MMTV/*Wnt1* mammary carcinomas [89], [90].

In aggregate these data suggest the MMTV/*Wnt1* strain as a potentially useful model of basal breast cancer in humans. The basal subtype has recently become a focus of intense concern because such tumors constitute a therapeutically intractable subset of breast cancers [91]–[93]. Since mammary stem cells have been hypothesized to reside in the basal layer, our observations also support the notion that Wnt1 may stimulate expansion of the mammary stem cell population [94], [95]. Intriguingly, the endogenous *Pea3* expression profiles that we have observed in mouse mammary gland (Figures 1 and 2) and in the colonic crypt (data not shown) are strikingly similar to those reported for the putative stem cell marker gene *Lgr5* [96], giving credence to the notion that *Pea3* expression may be enriched in stem cell compartments.
